# A Human Model of Small Fiber Neuropathy to Study Wound Healing

**DOI:** 10.1371/journal.pone.0054760

**Published:** 2013-01-31

**Authors:** Ben M. W. Illigens, Christopher H. Gibbons

**Affiliations:** Department of Neurology, Beth Israel Deaconess Medical Center, Harvard Medical School, Boston, Massachusetts, United States of America; University of Würzburg, Germany

## Abstract

The aim of this study was to develop a human model of acute wound healing that isolated the effects of small fiber neuropathy on the healing process. Twenty-five healthy subjects had the transient receptor vanilloid 1 agonist capsaicin and placebo creams topically applied to contralateral areas on the skin of the thigh for 48 hours. Subjects had shallow (1.2 millimeter) and deep (>3 millimeter) punch skin biopsies from each thigh on days 1 and 14. Biopsy wound healing was monitored photographically until closure. Intra-epidermal and sweat-gland nerve fiber densities were measured for each biopsy. Shallow wounds in capsaicin-treated sites healed more slowly than in placebo treated skin with biopsies taken on day 1 (*P*<0.001) and day 14 (*P*<0.001). Deep biopsies in the capsaicin and placebo areas healed at similar rates at both time points. Nerve fiber densities were reduced only in capsaicin treated regions (*P*<0.01). In conclusion, topical application of capsaicin causes a small fiber neuropathy and is associated with a delay in healing of shallow, but not deep wounds. This novel human model may prove valuable in the study of wound healing in patients with neuropathy.

## Introduction

Small fiber neuropathy is common in diseases such as diabetes and leads to impaired neurogenic inflammation and wound-healing capacity [1], [2]. Damage to small sensory nerve fibers leads to reduced axon-reflex vasodilation (mediated by nociceptive C-fibers), which is a critical component to neurogenic inflammation [3]–[5]. However, the mechanism by which dysfunction of small sensory nerve fibers contributes to reduced wound-healing capacity is not fully understood [6].

There are no studies in humans that specifically examine the effect of small fiber neuropathy on wound healing. Most studies of wound healing in animals are performed with other comorbid risk factors [7]–[12]. It is widely accepted that neuropathy increases the risk of ulcer development in humans but the specific effects of neuropathy on wound healing are less clear. A prior study investigating the healing of acute biopsy wounds did not find any difference in healing rates between healthy subjects and patients with diabetes [1], although other studies have demonstrated delayed vascular and neural repair in humans with diabetes [13], [14]. Clinically, we have observed delayed healing of skin biopsies in some patients with neuropathy, but this has not been studied systematically.

In cutaneous tissue repair, re-epithelialization occurs through migration of keratinocytes from free wound edges [15]. In partial-thickness wounds, additional sources for keratinocytes are dermal adnexal structures, such as hair follicles and sweat glands, which remain in the wound bed. These adnexal structures can contribute to nearly half of the re-epithelialization process [16]–[21]. This is in contrast to deeper (full-thickness) wounds where hair and other adnexal structures no longer remain and wound healing is therefore delayed [16]–[21]. The cutaneous adnexal structures in the dermal layer are innervated by small sensory and autonomic nerve fibers, and damage to these nerve fibers might attenuate the contribution of dermal structures to the wound healing process [22]–[25].

We hypothesized that an injury to small sensory and autonomic nerves in otherwise healthy individuals would delay healing of partial but not full-thickness wounds. We theorized that disruption of local axon-reflexes using a chemical axotomy model would prevent stimulation of keratinocytes from adnexal structures during tissue injury, thereby delaying wound healing. In order to test this hypothesis, we designed a novel human model of wound healing to study the effects of small fiber neuropathy, with and without cutaneous adnexal structure input, on the healing process. We topically applied the transient receptor vanilloid 1 agonist capsaicin to the skin of healthy subjects to cause degeneration of cutaneous nociceptive and autonomic nerve fibers [14], [26], [27]. We performed partial and full-thickness skin biopsies in the capsaicin and placebo regions at different time points to create cutaneous wounds, monitored by high-resolution photography, to determine the effects of a small fiber neuropathy on wound healing rates.

## Methods

### Subjects

Twenty-seven healthy subjects were recruited for the study. Subjects were excluded from participation if they had evidence by history or exam of neuropathy, peripheral vascular disease, a history of medical disease, current use of medications (except oral contraceptives), tobacco or excessive alcohol use.

### Ethics Statement

The Institutional Review Board of the Beth Israel Deaconess Medical Center approved the study. All subjects signed an informed consent.

### 48-hour Capsaicin Protocol

After screening, all subjects had an occlusive bandage containing 2.4 g of 0.1% capsaicin cream (Chattem Inc., Chattanooga, TN) or placebo cream (Johnson & Johnson, New Brunswick, NJ) applied to a 4×4 inch region of skin. Capsaicin and placebo creams were applied to corresponding areas on the mid-anterior thigh of opposite legs. Subjects were selected by block randomization to have placebo cream applied to one thigh and capsaicin cream to the other. The bandages remained on the skin for 24 hours with reapplication of both capsaicin and placebo on day 2 for another 24 hours [14], [27].

### Testing Protocol

After removal of the capsaicin and placebo creams, subjects underwent quantitative sensory testing, laser-Doppler flowmetry and quantitative sudomotor axon reflex testing within the demarcated area of both legs. Subjects then underwent two 3-mm punch skin biopsies (partial-thickness (shallow) and full-thickness (deep)) in the capsaicin and placebo treated areas respectively (defined as the day 1 biopsies). Two weeks later, subjects had another shallow and deep 3-mm punch skin biopsy taken adjacent to the original biopsies (defined as day 14 biopsies), totaling 8 biopsies per participant. Each biopsy site was monitored with high-resolution digital images 3 days per week to record healing rate until complete wound closure. All tests were repeated on days 7, 14, 21 and 28 after removal of capsaicin/placebo creams.

### Biopsy Protocol

**Figure 1:**
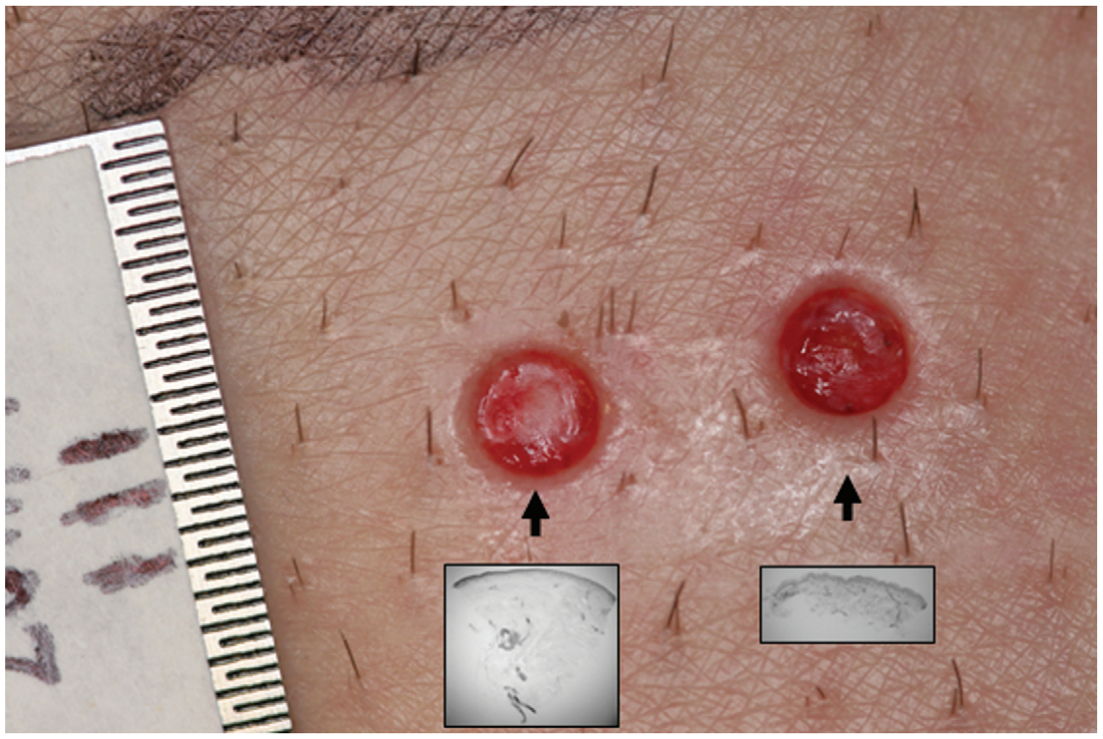
Skin biopsies as a model of wound healing. Three millimeter punch skin biopsies from the thigh with a ruler used as an internal standard measurement. The shallow biopsy (shown in subset) on the right contains pieces of hair follicles seen within the wound bed. The deep biopsy (shown in subset) is on the left and contains no remnants of adnexal structures within the wound bed.

Punch skin biopsies (3-mm diameter) were obtained after local anesthesia using 0.2 ml of 2% lidocaine with epinephrine (1∶10,000, Hospira, Inc., Lake Forest, IL). Shallow and deep skin biopsies were taken from each site. A shallow (partial-thickness) punch skin biopsy, 0.9–1.5 millimeters deep, was obtained leaving residual dermal structures in the wound bed. Shallow biopsies were performed by allowing the punch biopsy tool to enter only 1–1.5 millimeters into the skin, followed by tissue removal using a scalpel running parallel to the epidermis. A deep (full-thickness) biopsy, 3.2–4.6 millimeters deep, was acquired using a punch biopsy depth of 4 millimeters with removal of all dermal structures within the biopsy area (Figure 1). The biopsy specimens served as the tissue source for structural analysis and the remaining wounds were monitored for healing.

### Skin Biopsy Analysis

Biopsy specimens were fixed in Zambonis solution, sectioned into 50 micrometer thick frozen sections and stained with protein gene product 9.5 (1∶1000, rabbit anti-PGP 9.5, CHEMICON International, Inc., Temecula, CA) as previously described [24], [28]. Biopsy depth was measured in all sectioned tissue. For all biopsies, intra-epidermal nerve fiber density (IENFD) was quantified by a physician blinded to treatment allocation and results were expressed as a linear density (number of fibers per millimeter) as previously described [28], [29]. Sweat gland nerve fiber density (SGNFD) was quantified using a manual unbiased stereologic technique as previously reported [22], [24]. Briefly, a microscope with a 6.2 megapixel camera (PixeLINK, Ontario, Canada) was used to image tissue sections at 20× magnification and pictures were transferred to Image Pro-Plus (Version 6.0, Media Cybernetics, Bethesda, MD). A standardized grid of circles (10 µm in diameter, spaced 50 µm apart horizontally, and offset 25 µm vertically) was placed over the sweat gland image. The number of nerve fibers intersecting the circles within the area of interest was counted. Results are reported as the percentage of total circles with nerve fiber crossing [22], [24].

### Wound Healing

**Figure 2:**
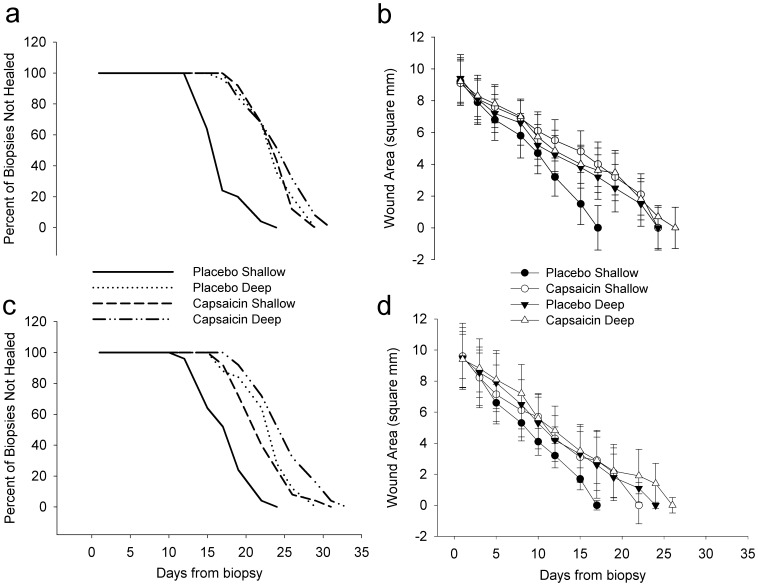
Rates of shallow and deep wound healing from capsaicin and placebo treated skin. Wound healing from day 1 (a & b) and day 14 (c & d) biopsies. Survival curves (a & c) are defined as the time from biopsy to wound closure (percent of biopsies not healed). The area of the open wound at each time point by biopsy type and treatment is shown for day 1 (b) and day 14 (d). Shallow biopsies from capsaicin treated areas healed more slowly than shallow biopsies from placebo treated areas (*P*<0.001 vs. placebo) on day 1 and day 14. There were no differences in capsaicin and placebo treated deep biopsies (*P* = 0.43 vs. placebo, day 1; *P* = 0.09 vs. placebo day 14). Shallow biopsies from capsaicin treated areas healed more quickly on day 14 (d) than on day 1 (b) (*P* = 0.03). Statistical significance is not displayed in these graphs.

Biopsy wound pictures were recorded with an 8.2 megapixel Canon EOS 30D (with attachment of a 100-mm Canon macro lens for magnification and a MR-14EX Canon Ring Lite for illumination; Canon USA Inc, Lake Success, NY). Images of each biopsy were taken 3 days per week (Monday, Wednesday, Friday) starting with the day of the biopsy. Each wound image contained a ruler as an internal standard (Figure 2). The area of the wound was measured by tracing the edge of the biopsy in Image Pro-Plus (Version 6.0, Media Cybernetics, Bethesda, MD) in triplicate by technicians blinded to treatment. The average result was reported; if variance in measurements exceeded 10%, the image tracing was repeated. Healing was defined as complete re-epithelialization or loss of the wound scab.

### Quantitative Sensory Testing

Testing included cold, heat, cold-pain and heat-pain perception thresholds at each visit. Testing was performed with ascending (heat) or descending (cold) methods of limits using standard protocols [30]. Thermal stimuli were applied using a 16×16 mm thermode (TSA II Neurosensory Analyzer, Medoc Ltd., Israel). The results of 4 individual trials for heat and cold, and 3 individual trials for heat-pain and cold-pain were averaged. Subject instructions were standardized with cueing cards. A temperature maximum of 52°C was set to prevent thermal injury [30].

### Quantitative Sudomotor Axon Reflex Testing

Quantitative sudomotor axon reflex testing was performed following the standard protocol with iontophoresis of acetylcholine solution (10% in sterile water) into the skin at 2 mA for 5 minutes [31]. Recordings were analyzed by area under the curve (AUC) for the first 15 minutes after iontophoresis.

### Laser-Doppler Flowmetry

The C-fiber mediated axon-reflex was measured using the laser-Doppler flowmetry technique after acetylcholine iontophoresis using standard protocols [32]. C-fiber mediated vasodilation was provoked by acetylcholine (Penta, Fairfield, NJ; 1% in sterile water) using anodal current of 0.4 mA for 140 seconds. The Periflux System 5000 laser-Doppler flowmeter (Perimed, Jarfalla, Sweden) using an 8-laser integrating probe measured cutaneous blood flow [32]. The percent change from baseline in response to acetylcholine iontophoresis was reported. Tests were performed at ambient room temperatures between 23.7°C and 25.6°C.

### Statistical Analysis

Data are presented as mean ± standard deviation. For the primary analysis, time (in days) to wound closure for capsaicin and placebo treated skin was analyzed by paired t-test, for shallow and deep wounds respectively. Survival curves were plotted to represent wound healing time, with survival defined as the period from biopsy to wound closure. Repeated measures ANOVA and post-hoc pairwise analysis with Bonferonni correction was performed for quantitative sensory testing, quantitative sweat testing and laser-Doppler flowmetry. Nerve fiber densities were compared by paired t-test. A two-sided *P* value <0.05 was used to define statistical significance for all data sets, with all results compared to placebo. Statistical data analysis was performed using SPSS v16.0 (IBM, Chicago, IL).

## Results

### Demographics

Twenty-five of 27 subjects completed the testing protocol. Two subjects withdrew prior to wound closure; their results are not included in the final analysis (data were within the range of all other subjects at the time of withdrawal; no complications were noted). The 25 healthy subjects who finished the study were aged 20–38 years with a mean age of 28.7 years. Eighteen of the subjects were female. Shallow (partial-thickness) skin biopsies had an average depth of 1.2 mm (±0.3 mm). Deep (full-thickness) biopsies had an average depth of 3.9 mm (±0.7 mm).

### Wound Healing

For day 1 biopsies, healing rates of shallow wounds in capsaicin treated sites were significantly delayed compared to placebo treated sites (24.4±2.7 days capsaicin vs. 17.5±2.8 days placebo, *P<*0.001; Figure 2). Deep biopsy wounds in the capsaicin and placebo areas healed at similar rates (25.1±3.8 days capsaicin vs. 24.2±3.3 days placebo, *P = 0.43*; Figure 2). For day 14 biopsies, healing rates of shallow wounds in capsaicin treated sites were significantly delayed compared to placebo treated sites (22.4±3.1 days capsaicin vs. 17.6±2.8 days placebo, *P*<0.001; Figure 2). Day 14 deep wounds in the capsaicin and placebo areas healed at similar rates (25.6±3.8 days capsaicin vs. 23.5±3.4 days placebo; *P = 0.09*; Figure 2). Shallow wounds healed more quickly on day 14 than on day 1 from capsaicin treated regions (*P* = 0.03; Figure 2) No differences in gender or location were noted in wound healing rates within groups.

### Nerve Fiber Density

**Figure 3:**
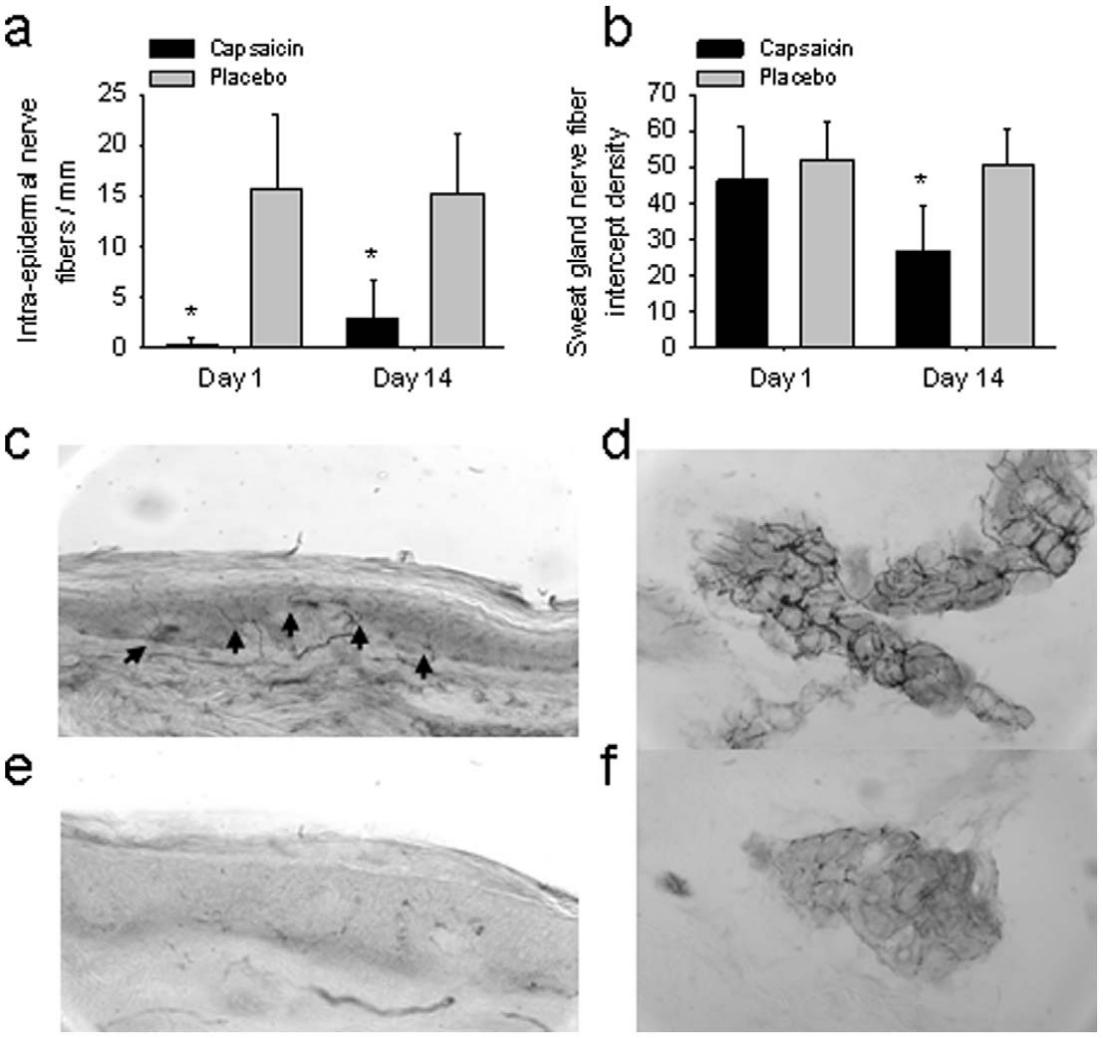
The intra-epidermal and sweat gland nerve fiber density in capsaicin and placebo treated skin. (a) The intra-epidermal nerve fiber density from biopsies taken on day 1 and 14 is shown (mean ± SD). *P<0.001 compared to control. (b) The sweat gland nerve fiber density from biopsies taken on day 1 and 14 is shown (mean ± SD). *P<0.01 compared to control. (c, e) Protein gene product 9.5 labeled intra-epidermal nerve fibers from day 14 biopsies in placebo (c) and capsaicin (d) treated skin. Black arrows denote intra-epidermal fibers in placebo treated skin (c), but are not seen in capsaicin treated skin (e). (d, f) Protein gene product 9.5 labeled sweat gland nerve fibers from day 14 biopsies in placebo (d) and capsaicin (f) treated skin.

In biopsy specimens obtained on day 1 from capsaicin treated areas, intra-epidermal nerve fiber density was significantly reduced compared to placebo (0.4±0.6 fibers/mm capsaicin vs. 15.7±7.3 fibers/mm placebo, *P*<0.001: Figure 3). In biopsies taken on day 14 from the capsaicin treated areas, intra-epidermal nerve fiber density was higher compared to day 1 but still reduced compared to placebo (3.0±3.6 fibers/mm capsaicin vs. 15.2±5.8 fibers/mm placebo, *P<*0.001: Figure 3). No difference was noted between intra-epidermal nerve fiber density from shallow and deep skin biopsies.

The sweat gland nerve fiber density was similar between deep (full thickness) biopsies from capsaicin and placebo treated sites on day 1 (46.3±14.9 intercept density capsaicin vs. 52.0±10.6 intercept density placebo, *P* = 0.68*:* Figure 3). The sweat gland nerve fiber density was reduced in deep biopsies from capsaicin treated areas on day 14 (26.7±12.8 intercept density capsaicin vs. 50.7±9.6 intercept density placebo, *P<*0.01: Figure 3). Sweat glands were not found within shallow (partial-thickness) biopsies.

### Tests of Nerve Fiber Function

**Figure 4:**
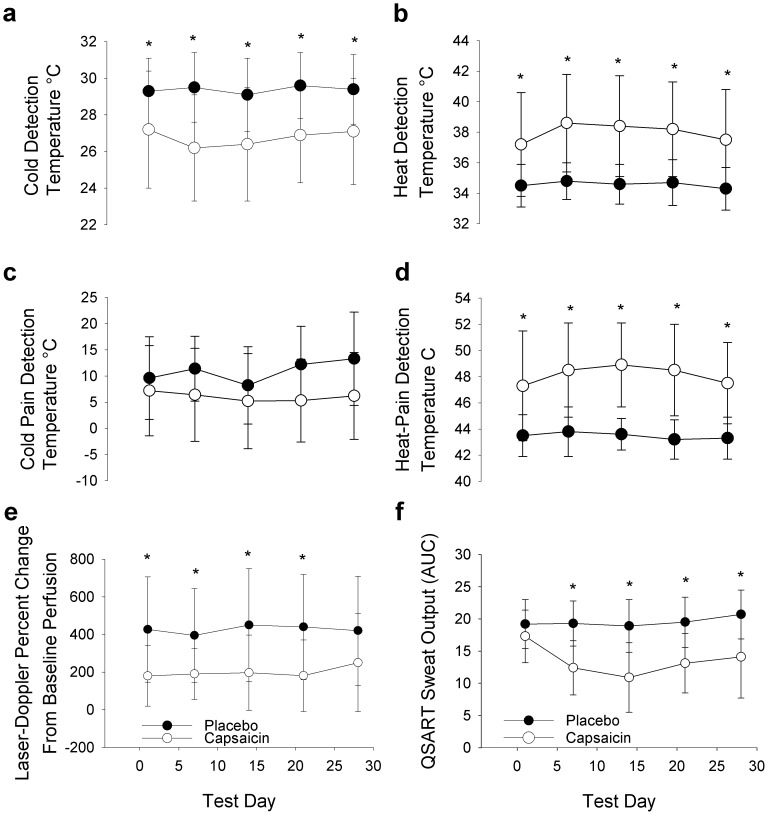
Neurophysiologic testing in capsaicin and placebo treated skin. The cold (a), heat (b), cold-pain (d), and heat-pain (d) detection thresholds are shown for each test visit for placebo (black circles) and capsaicin (open circles) treated regions. Cold detection thresholds were lower for every test day in the capsaicin treated side. Heat and heat-pain detection thresholds were higher for every test day on the capsaicin treated side. No significant differences were noted in cold-pain detection thresholds. Blood flow measured by laser-Doppler flowmetry is shown in (e) for placebo (black circles) and capsaicin (open circles) treated regions. Axon-reflex mediated blood flow was reduced in the capsaicin treated region compared to placebo treated region on all test days except day 28. Sweat volume measured by the quantitative sudomotor axon reflex test (QSART) is shown in (f). Sweat output was reduced in the capsaicin treated region compared to the placebo treated region on all test days except day 1. AUC = area under the curve. *P<0.05.

In the capsaicin treated skin, detection thresholds were lower for cold, higher for heat and higher for heat-pain on all testing days compared to placebo treated skin (1, 7, 14, 21 and 28; *P*<0.01; Figure 4a–d). Cold-pain detection thresholds were not significantly different between groups (*P* = 0.28). There was a decrease in the nociceptive axon-reflex mediated response to acetylcholine iontophoresis in capsaicin treated skin as measured by laser-Doppler flowmetry on test days 1, 7, 14 and 21 (*P*<0.05; Figure 4e). There was no statistically significant difference in laser-Doppler flowmetry on day 28 between capsaicin and placebo groups. There was a reduction in sweat output in capsaicin treated compared to placebo treated skin on day 7, day 14, day 21 and day 28, but not day 1 (*P*<0.01; Figure 4f).

## Conclusions

In this study, we report a novel human model of acute wound healing. We, and others, have previously shown that topical application of the TRPV1 agonist capsaicin causes both structural and functional changes to cutaneous sensory and autonomic nerve fibers [14], [27]. In the current investigation, we demonstrate that topical application of capsaicin, resulting in a small fiber neuropathy, delays healing of partial-thickness wounds by more than 30% compared to wounds in normal skin. However, no delay in healing occurs in deeper wounds with adnexal structures removed.

After 48 hour occlusive capsaicin treatment, there was near-complete loss of intra-epidermal nerve fibers and reduction in sweat gland nerve fiber density with functional changes to quantitative sensory testing, quantitative sudomotor testing and axon-reflex vasodilation, consistent with several prior studies [14], [27]. We found that shallow wounds taken 1 day after capsaicin application heal at the same rate as full-thickness wounds that have all adnexal structures removed. Shallow biopsies taken 14 days after capsaicin treatment also healed more slowly than placebo biopsies, thus demonstrating that the delay in wound healing from day 1 biopsies is not solely a consequence of local neuropeptide depletion or a transient effect of capsaicin pre-treatment on local signaling or cellular migration [33]. Capsaicin pretreatment had no effect on full-thickness wound healing rates and results did not differ from placebo.

A number of possible mechanisms for the delayed wound healing seen in our capsaicin treated biopsies are suggested by animal studies. After cutaneous injury, recruited keratinocytes migrate from the wound edges into the wound bed while keratinocytes in adnexal structures contribute to nearly half of the re-epithelialization area in partial thickness wounds [16]. Animal studies have also shown a delay in wound healing when dermal structures are removed [34]. A recent study in rats demonstrated that capsaicin denervation impairs hair follicle stem cell migration leading to delays in wound healing [35]. These results are consistent with our findings where full thickness biopsies heal more slowly than partial thickness biopsies from placebo treated skin that still contain adnexal structures in the wound bed.

A recent study of the effects of both chemical and excisional axotomy demonstrates that vascular regrowth after tissue injury precedes axonal regrowth [13]. In addition, delayed vascular regrowth is seen in patients with diabetes. Our study compared placebo and capsaicin treated sites within the same individual to avoid confounding differences in baseline vascular density on wound healing. Our functional data (sweat, sensory and axon-reflex vasodilation) suggest that capsaicin denervation effectively disrupts the neurogenic axon reflex. However, we cannot distinguish the neurovascular effects on wound healing from the neural effects on other dermal appendages using the capsaicin model. Nonetheless, our data strongly suggest that neuropathy may predispose to delayed cutaneous tissue repair. Although we did not identify the mechanism of delayed healing in our study, a recent report using chemical axotomy in rats supports our theory that impaired stem cell migration from dermal adnexal structures may be secondary to disruption of local neural reflexes [35].

There are several limitations to our study. Capsaicin acts widely across many cell types that possess TRPV1 receptors. Although capsaicin is not known to cause damage to endothelial progenitor cells, it is possible that the application of capsaicin does reduce progenitor cell migration, nerve signaling or other actions involved in wound healing. Capsaicin may also alter keratinocyte proliferation and this study is unable to distinguish acute capsaicin effects on healing from neuropathy specific effects, although we would expect deep biopsies from capsaicin treated regions to heal more slowly if there was a significant effect on keratinocyte proliferation or migration. However, shallow skin biopsies taken 2 weeks after capsaicin application still showed delayed wound healing, a finding that cannot be explained by transient effects of capsaicin. The evidence of neural regrowth on day 14, seen by increasing numbers of intra-epidermal nerve fibers, demonstrate that the acute effects of capsaicin have resolved. Another limitation is that topical capsaicin does not penetrate deeply into the dermal layer. Our prior studies involving topical capsaicin application suggest a partial reduction of sudomotor and vasomotor fibers, but not complete loss as seen with epidermal nerve fibers [27]. It is unclear if the partial loss of nerve fibers in deeper tissues is secondary to lower densities of TRPV1 channels on non-nociceptive nerve fibers or diminished penetration of capsaicin to deeper tissues. Most individuals with small fiber neuropathy are older while our study included primarily younger individuals. We intentionally chose a homogenous population to reduce the number of potential variables to consider in the wound healing process. Additional study of healing rates in an older population would provide valuable information about the effects of age on healing rates.

We report a unique method that may isolate the effects of a small nerve fiber injury on wound healing. The technique creates a standardized nerve injury that is reproducible, transient and can study both healthy subjects and patients with disease without any long-term consequences. Many animal models of wound healing are available, but often do not reflect the human condition because rodent models of cutaneous injury typically heal by contracture, while humans heal by re-epithelialization. In order to accelerate discovery of novel therapies for wound healing, and to translate them into clinical practice, it is imperative that effective human models exist.

Our study demonstrates that application of topical capsaicin, with an associated small fiber neuropathy, causes a delay in healing of shallow wounds by 30% or more, leading to healing rates similar to those of deep wounds. The effects of topical capsaicin on wound healing appear to last at least 2 weeks beyond the time of application. Our study suggests that small nerve fibers may be a relevant contributor in the pathogenesis of acute wound healing, but only when dermal structures remain intact - a scenario that reflects the majority of acute superficial wounds. Our protocol includes prolonged application of capsaicin in an occlusive dressing, a technique that increases the delivered dose of capsaicin beyond clinical guidelines. Our findings may not apply to the more widespread clinical use of capsaicin as clinical trials have not reported delayed wound healing as a complication [36]–[38]. Nonetheless, we suggest that capsaicin, widely advocated for treatment of neuropathic pain, be used with caution in patients that are at risk for or already have wound healing impairment.

## References

[1] Krishnan ST, Quattrini C, Jeziorska M, Malik RA, Rayman G (2007) Neurovascular factors in wound healing in the foot skin of type 2 diabetic subjects. Diabetes Care 30: 3058–3062. 17898089 10.2337/dc07-1421

[2] Adler AI, Boyko EJ, Ahroni JH, Smith DG (1999) Lower-extremity amputation in diabetes. The independent effects of peripheral vascular disease, sensory neuropathy, and foot ulcers. Diabetes Care 22: 1029–1035. 10388962 10.2337/diacare.22.7.1029

[3] Hamdy O, Abou-Elenin K, LoGerfo FW, Horton ES, Veves A (2001) Contribution of nerve-axon reflex-related vasodilation to the total skin vasodilation in diabetic patients with and without neuropathy. Diabetes Care 24: 344–349. 11213890 10.2337/diacare.24.2.344

[4] Caselli A, Rich J, Hanane T, Uccioli L, Veves A (2003) Role of C-nociceptive fibers in the nerve axon reflex-related vasodilation in diabetes. Neurology 60: 297–300. 12552048 10.1212/01.wnl.0000040250.31755.f9

[5] Kramer HH, Schmelz M, Birklein F, Bickel A (2004) Electrically stimulated axon reflexes are diminished in diabetic small fiber neuropathies. Diabetes 53: 769–774. 14988263 10.2337/diabetes.53.3.769

[6] Boyko EJ, Ahroni JH, Stensel V, Forsberg RC, Davignon DR, et al (1999) A prospective study of risk factors for diabetic foot ulcer. The Seattle Diabetic Foot Study. Diabetes Care 22: 1036–1042. 10388963 10.2337/diacare.22.7.1036

[7] Chan FC, Kennedy C, Hanson RP, O’Sullivan B, Kelly J, et al (2007) Topical diphenylhydantoin sodium can improve healing in a diabetic incisional animal wound model. JWoundCare 16: 359–363. 10.12968/jowc.2007.16.8.27858 17927083

[8] O’Sullivan JB, Hanson R, Chan F, Bouchier-Hayes DJ (2010) Tight glycaemic control is a key factor in wound healing enhancement strategies in an experimental diabetes mellitus model. IrJMedSci. 10.1007/s11845-010-0630-z 21110137

[9] Thomson SE, McLennan SV, Hennessy A, Boughton P, Bonner J, et al (2010) A novel primate model of delayed wound healing in diabetes: dysregulation of connective tissue growth factor. Diabetologia 53: 572–583. 20091023 10.1007/s00125-009-1610-6

[10] Jacobsen JN, Steffensen B, Hakkinen L, Krogfelt KA, Larjava HS (2010) Skin wound healing in diabetic beta6 integrin-deficient mice. APMIS 118: 753–764. 20854469 10.1111/j.1600-0463.2010.02654.x PMC2964129

[11] Michaels J, Churgin SS, Blechman KM, Greives MR, Aarabi S, et al (2007) db/db mice exhibit severe wound-healing impairments compared with other murine diabetic strains in a silicone-splinted excisional wound model. WoundRepair Regen 15: 665–670. 10.1111/j.1524-475X.2007.00273.x 17971012

[12] Laing T, Hanson R, Chan F, Bouchier-Hayes D (2010) Effect of pravastatin on experimental diabetic wound healing. JSurgRes 161: 336–340. 10.1016/j.jss.2009.01.024 20031169

[13] Ebenezer GJ, O’Donnell R, Hauer P, Cimino NP, McArthur JC, et al (2011) Impaired neurovascular repair in subjects with diabetes following experimental intracutaneous axotomy. Brain 134: 1853–1863. 21616974 10.1093/brain/awr086 PMC3140859

[14] Polydefkis M, Hauer P, Sheth S, Sirdofsky M, Griffin JW, et al (2004) The time course of epidermal nerve fibre regeneration: studies in normal controls and in people with diabetes, with and without neuropathy. Brain 127: 1606–1615. 15128618 10.1093/brain/awh175

[15] Martin P (1997) Wound healing–aiming for perfect skin regeneration. Science 276: 75–81. 9082989 10.1126/science.276.5309.75

[16] Li J, Chen J, Kirsner R (2007) Pathophysiology of acute wound healing. ClinDermatol 25: 9–18. 10.1016/j.clindermatol.2006.09.007 17276196

[17] Fuchs E (1998) Beauty is skin deep: the fascinating biology of the epidermis and its appendages. Harvey Lect 94: 47–77. 11070952

[18] Ordman LJ, Gillman T (1966) Studies in the healing of cutaneous wounds. I. The healing of incisions through the skin of pigs. ArchSurg 93: 857–882. 10.1001/archsurg.1966.01330060001001 5954325

[19] Ordman LJ, Gillman T (1966) Studies in the healing of cutaneous wounds. 3. A critical comparison in the pig of the healing of surgical incisions closed with sutures or adhesive tape based on tensile strength and clinical and histological criteria. ArchSurg 93: 911–928. 10.1001/archsurg.1966.01330060055003 5333550

[20] Ordman LJ, Gillman T (1966) Studies in the healing of cutaneous wounds. II. The healing of epidermal, appendageal, and dermal injuries inflicted by suture needles and by the suture material in the skin of pigs. ArchSurg 93: 883–910. 10.1001/archsurg.1966.01330060027002 5333549

[21] Schneider MR, Werner S, Paus R, Wolf E (2008) Beyond wavy hairs: the epidermal growth factor receptor and its ligands in skin biology and pathology. AmJPathol 173: 14–24. 10.2353/ajpath.2008.070942 PMC2438281 18556782

[22] Gibbons CH, Illigens BM, Wang N, Freeman R (2010) Quantification of sudomotor innervation: a comparison of three methods. Muscle Nerve 42: 112–119. 20544913 10.1002/mus.21626 PMC3048308

[23] Dabby R, Vaknine H, Gilad R, Djaldetti R, Sadeh M (2007) Evaluation of cutaneous autonomic innervation in idiopathic sensory small-fiber neuropathy. JPeripherNervSyst 12: 98–101. 10.1111/j.1529-8027.2007.00128.x 17565534

[24] Gibbons CH, Illigens BM, Wang N, Freeman R (2009) Quantification of sweat gland innervation: a clinical-pathologic correlation. Neurology 72: 1479–1486. 19398703 10.1212/WNL.0b013e3181a2e8b8 PMC2677479

[25] Wang N, Gibbons CH, Freeman R (2011) Novel immunohistochemical techniques using discrete signal amplification systems for human cutaneous peripheral nerve fiber imaging. JHistochemCytochem 59: 382–390. 10.1369/0022155410396931 PMC3201146 21411809

[26] Nolano M, Simone DA, Wendelschafer-Crabb G, Johnson T, Hazen E, et al (1999) Topical capsaicin in humans: parallel loss of epidermal nerve fibers and pain sensation. Pain 81: 135–145. 10353501 10.1016/s0304-3959(99)00007-x

[27] Gibbons CH, Wang N, Freeman R (2010) Capsaicin induces degeneration of cutaneous autonomic nerve fibers. AnnNeurol 68: 888–898. 10.1002/ana.22126 PMC3057686 21061393

[28] Gibbons CH, Griffin JW, Polydefkis M, Bonyhay I, Brown A, et al (2006) The utility of skin biopsy for prediction of progression in suspected small fiber neuropathy. Neurology 66: 256–258. 16434668 10.1212/01.wnl.0000194314.86486.a2

[29] Lauria G, Hsieh ST, Johansson O, Kennedy WR, Leger JM, et al (2010) European Federation of Neurological Societies/Peripheral Nerve Society Guideline on the use of skin biopsy in the diagnosis of small fiber neuropathy. Report of a joint task force of the European Federation of Neurological Societies and the Peripheral Nerve Society. EurJNeurol 17: 903–909. 10.1111/j.1468-1331.2010.03023.x 20642627

[30] Yarnitsky D (1997) Quantitative sensory testing. Muscle Nerve 20: 198–204. 9040659 10.1002/(sici)1097-4598(199702)20:2<198::aid-mus10>3.0.co;2-#

[31] Low PA, Caskey PE, Tuck RR, Fealey RD, Dyck PJ (1983) Quantitative sudomotor axon reflex test in normal and neuropathic subjects. AnnNeurol 14: 573–580. 10.1002/ana.410140513 6316835

[32] Berghoff M, Kathpal M, Kilo S, Hilz MJ, Freeman R (2002) Vascular and neural mechanisms of ACh-mediated vasodilation in the forearm cutaneous microcirculation. JApplPhysiol 92: 780–788. 10.1152/japplphysiol.01167.2000 11796692

[33] Lai X, Wang Z, Wei L, Wang L (2002) Effect of substance P released from peripheral nerve ending on endogenous expression of epidermal growth factor and its receptor in wound healing. Chin JTraumatol 5: 176–179. 12034083

[34] Miller SJ, Burke EM, Rader MD, Coulombe PA, Lavker RM (1998) Re-epithelialization of porcine skin by the sweat apparatus. JInvest Dermatol 110: 13–19. 9424080 10.1046/j.1523-1747.1998.00087.x

[35] Martinez-Martinez E, Galvan-Hernandez CI, Toscano-Marquez B, Gutierrez-Ospina G (2012) Modulatory role of sensory innervation on hair follicle stem cell progeny during wound healing of the rat skin. PLoS One 7: e36421. 22574159 10.1371/journal.pone.0036421 PMC3344885

[36] Low PA, Opfer-Gehrking TL, Dyck PJ, Litchy WJ, O’Brien PC (1995) Double-blind, placebo-controlled study of the application of capsaicin cream in chronic distal painful polyneuropathy. Pain 62: 163–168. 8545141 10.1016/0304-3959(94)00261-C

[37] Biesbroeck R, Bril V, Hollander P, Kabadi U, Schwartz S, et al (1995) A double-blind comparison of topical capsaicin and oral amitriptyline in painful diabetic neuropathy. AdvTher 12: 111–120. 10150323

[38] Simpson DM, Brown S, Tobias J (2008) Controlled trial of high-concentration capsaicin patch for treatment of painful HIV neuropathy. Neurology 70: 2305–2313. 18541884 10.1212/01.wnl.0000314647.35825.9c

